# Autoimmune-Associated Vasculitis Presenting as Ischemic Stroke With Hemorrhagic Transformation: A Case Report and Literature Review

**DOI:** 10.7759/cureus.10403

**Published:** 2020-09-12

**Authors:** Salil Uppal, Sandeep Goel, Baljit Randhawa, Ankush Maheshwary

**Affiliations:** 1 Neurology, Uppal Neuro Hospital (UNH), Amritsar, IND; 2 Medicine, Government Medical College, Amritsar, IND

**Keywords:** hemorragic stroke, wegener's granulomatosis, ischemic stroke, young onset stroke, neurological autoimmune disorders, autoimmune stroke, antineutrophil cytoplasmic antibody (anca) associated vasculitis (aav), central nervous system vasculitis, small vessel vasculitis

## Abstract

Autoimmune-associated vasculitis is related to conditions like granulomatosis with polyangiitis (GPA) and eosinophilic polyangiitis with granulomatosis (EGPA), among many others. An unlikely scenario is patients with the above conditions presenting with ischemic strokes before any renal or pulmonary pathology. These conditions are associated with increased antineutrophillic cytoplasmic antibodies (C-ANCA) levels in the blood, and its decline after treatment is directly proportional to the recovery of the patient. We present a case of a previously healthy 38-year-old male patient who presented with acute/subacute ischemic stroke with elevated C-ANCA levels; his MRI brain images revealed multiple posterior circulation infarcts with hemorrhagic transformation. With pulse steroid therapy, he had significant improvement in neurological functions. This case report highlights the importance of maintaining a high degree of suspicion and providing early treatment for autoimmune strokes in young patients with no clear etiology for such a presentation.

## Introduction

Central nervous system (CNS) involvement in Wegener's granulomatosis (WG) often occurs in advanced stages with a wide range of latency period that ranges from five months to seven years; however, it occurs as the initial presentation in 1-2.5% cases [1]. The Trial of ORG 10172 in Acute Stroke Treatment (TOAST) [2] classifies stroke based on etiology into the following categories: 1. large artery atherosclerosis; 2. cardioembolism; 3. small vessel disease; 4. stroke of other determined etiology; and 5. stroke of undetermined etiology.

About 30 years ago, Davies et al. discovered antibodies typically staining ethanol-fixed neutrophils in a diffuse cytoplasmic manner. These antibodies were found to be against proteinase 3 (PR3) enzymes, and hence the terms anti-PR3 and antineutrophillic cytoplasmic antibodies (C-ANCA) are used interchangeably [3]. The group of diseases associated with the presence of C-ANCA in the blood is called ANCA-associated vasculitis (AAV). It includes WG, eosinophilic polyangiitis with granulomatosis (EGPA), microscopic polyangiitis (MPA), and pauci-immune glomerulonephritis [4]. Many studies have shown the CNS involvement in AAV, but with stroke, this association has not been well established, and hence it is a diagnostic dilemma for neurologists. Mourguet et al. were the first to prove a statistically increased incidence of cerebrovascular accidents (CVA) in patients with AAV, with a relative risk of 1.20 (95% CI: 0.98-1.48) [4]. We present a case of a middle-aged man who presented in a semiconscious state with a history of vertigo, which had led to an incidence of a fall. On brain imaging, he had multiple infarcts with hemorrhagic transformation but without any known risk factor. After ruling out all the possible causes of the stroke, high titers of C-ANCA antibodies were found in his blood in the absence of typical signs of vasculitis like renal or respiratory involvement. We hope this case report will shed some light on an unlikely presentation of autoimmune vasculitis and, simultaneously, a unique etiology associated with stroke.

## Case presentation

The patient was a 38-year-old male with no history of diabetes, hypertension, or drug abuse. He had complaints of fever for a month, followed by sudden-onset vertigo and a falling episode. Soon after the event, he had developed weakness of the left side of the body, resulting in a semiconscious state in which he was brought to the hospital.

**Figure 1:**
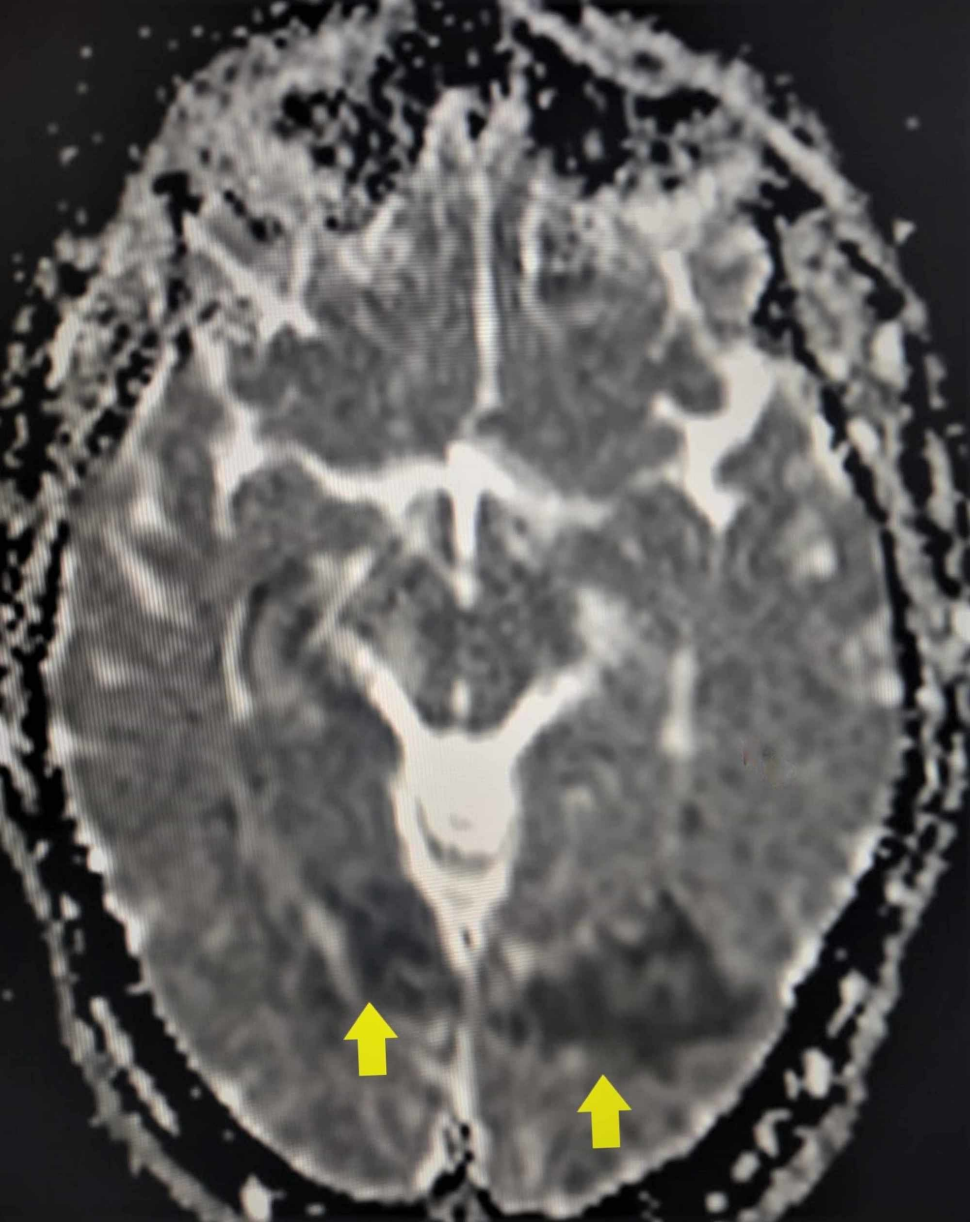
DW/ADC MRI sequence The image shows areas of patchy diffusion restriction (yellow arrows) MRI: magnetic resonance imaging; DW: diffusion-weighted; ADC: apparent diffusion coefficient

On initial presentation, the patient had a Glasgow Coma Scale (GCS) score of 11 [E3 (eye opening to command) V3 (inappropriate speech) M5 (localizing to painful stimulus)]. He had a body mass index (BMI) of 28 kg/m^2^ (normal range: 18.5-24.9 kg/m^2^), a temperature of 100 °F (normal value: 98.6 °F), and his blood pressure (BP) was within normal limits (WNL). Bilateral air entry was equal, and heart sounds were WNL. His power on the left side of the body was 0/5 on both upper limb (UL) and lower limb (LL), and that on the right side was 5/5. Both the pupils were equal (0.5 cm) and reactive to light. He had no neck stiffness, and Brudzinski and Kernig's signs were negative. Immediately after the presentation, suspecting a CVA, the patient was taken for an MRI scan, which showed multiple areas of hyperintensities on T2/fluid-attenuated inversion recovery (FLAIR) images in the occipito-parieto-temporal regions bilaterally and also involving respective thalamus, suggestive of acute to subacute infarcts (Figure 1). His National Institute of Health Stroke Scale (NIHSS) score was 10 and the Modified Rankin Scale (MRS) score was 5 on presentation.

Based on the preliminary presentation, the patient was managed conservatively. Recombinant tissue-plasminogen activator (rTPA) was contraindicated due to delayed presentation and unclear etiology of his condition. Given his demographic and history, encephalitis and meningitis were considered as differentials. Due to the lack of digital subtraction angiography (DSA), a CT angiography (CTA) was performed instead, which showed no significant pathology. His routine blood biochemistry done on the same day showed blood glucose levels of 200 mg/dl (normal value: ≤200 mg/dl), deranged prothrombin time index (PTI), erythrocyte sedimentation rate (ESR) of 48 mm/hour (normal range: 0-16 mm/hour), platelet count of 4.65 lakhs/cumm (normal range: 1.3-4.50 lakhs/cumm), and total leukocyte count (TLC) of 11,700/cumm (normal range: 4,000-11,000/cumm). Cerebrospinal fluid (CSF) analysis showed glucose level of 118 mg/dl (normal range: 40-80 mg/dl), proteins of 68 mg/dl (normal range: 20-40 mg/dl), and lymphocytes of 80/cumm (normal range: 0-6/cumm); adenosine deaminase (ADA) was negative as was acid-fast bacillus (AFB) stain.

On the second day, an echocardiogram was done considering an embolic phenomenon, which came out normal; yet he was fixed with a 24-hour Holter monitor, which showed no fibrillation or arrhythmias. A complete autoimmune panel was ordered on the third day in which indirect fluorescence showed C-ANCA on ethanol-fixed human neutrophils quantifying at 68.8 U/ml (normal value: <20 U/ml). Anti-neutrophil antibody (ANA) titers were not raised at the time (1:80). The antibodies against myeloperoxidase (P-ANCA) were also negative. His chest X-ray showed no pulmonary nodules or signs of hilar lymphadenopathy.

**Figure 2:**
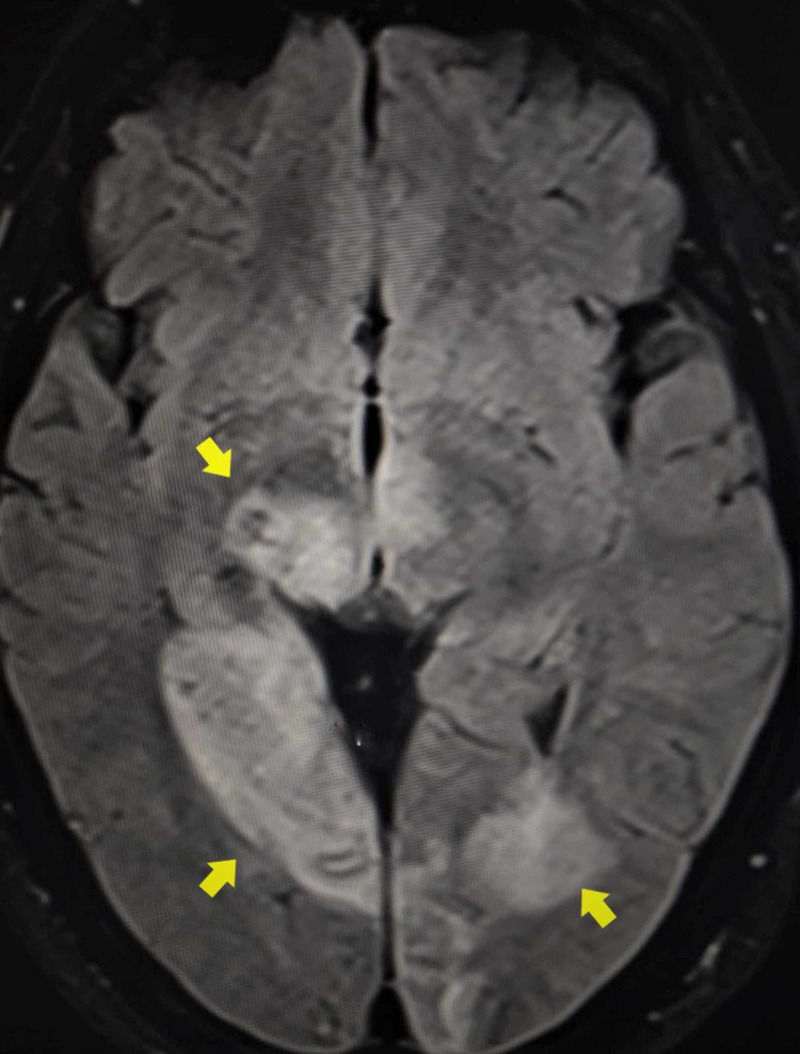
FLAIR sequence of MRI The image shows areas of hyperintensities in the bilateral occipito-parietal-temporal regions and bilateral thalamus (yellow arrows) MRI: magnetic resonance imaging; FLAIR: fluid-attenuated inversion recovery

**Figure 3:**
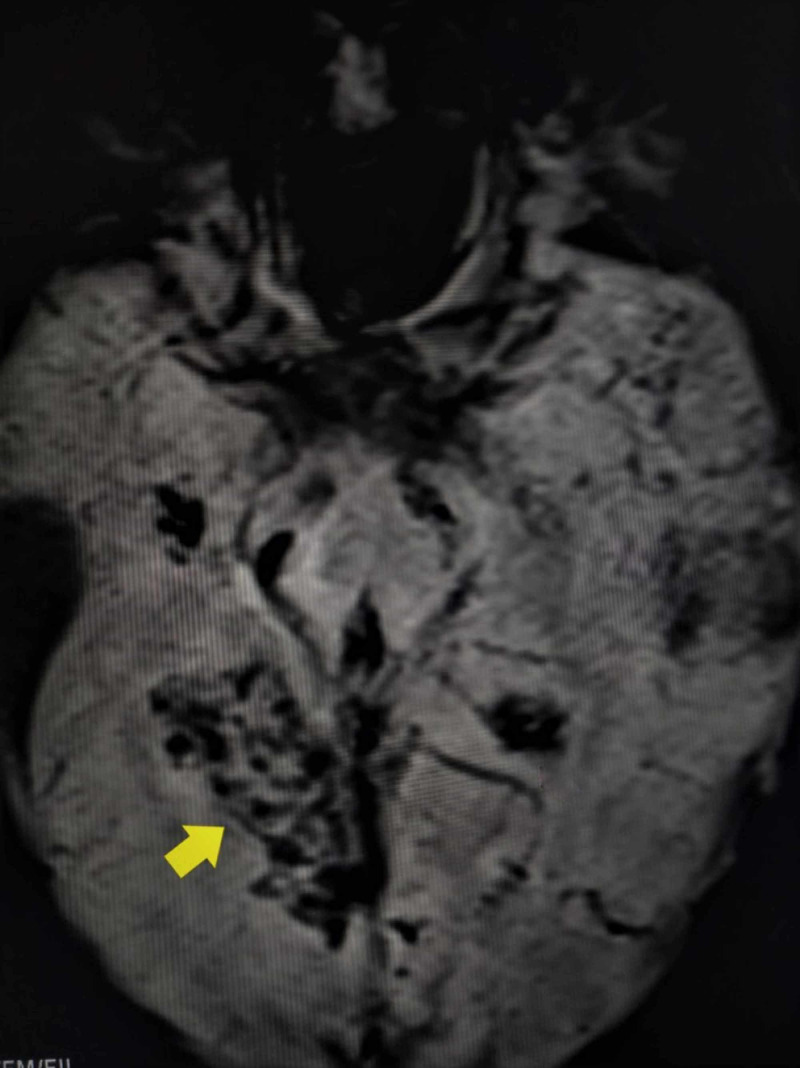
GRE sequence of CEMRI The image shows a blooming (yellow arrow) pattern CEMRI: contrast-enhanced magnetic resonance imaging; GRE: gradient echo

On the fourth day of hospitalization, the patient was again taken for a contrast-enhanced MRI (CEMRI). It showed altered signal with hyperintensities in the areas mentioned earlier on T2/FLAIR images (Figure 2) and blooming on gradient echo (GRE) sequences suggestive of hemorrhagic transformation superimposed on an already present acute to subacute infarct (Figure 3). Due to the raised levels of C-ANCA, he was immediately given steroid pulse therapy, which was initiated with 750 mg/day methylprednisolone intravenously for five days, and he was otherwise managed conservatively.

The patient recovered significantly with a gradual return of consciousness and he partially regained the power in his limbs (3/5 in UL and 3/5 in LL). The delayed presentation to the hospital led to only a partial recovery in the limbs. Methylprednisolone was gradually tapered and replaced with pulse cyclophosphamide therapy and oral prednisolone at 0.5 mg/kg/day. He was provided with assisted physical rehabilitation multiple times in a day, which was continued after that.

## Discussion

In a study conducted in the early 90s, Nishino et al. discovered that among the 324 patients diagnosed with WG, 33.6% developed neurological complications [5]. Among them, peripheral neuropathy was the most common complication, followed by cranial mononeuropathy and seizures. Almost 11% of the patients had infarction related to vasculitis [5]. Although our patient had high C-ANCA levels in his blood, he did not fit the criteria for either WG or EGPA according to the American College of Rheumatology classification. On initial presentation, he neither had upper respiratory symptoms nor did he have asthma or eosinophilia, as seen in EGPA [6].

Accelerated atherosclerosis has been emerging as an essential aspect of AAV in recent years. Studies have shown that increased cytokines production, especially Th17 and Th1, and lipid accumulation, proceeding to arterial inflammation, is associated with subclinical atherosclerosis [1]. Drachman et al. have described three main pathogenic mechanisms for C-ANCA-related CNS disease. The first mechanism is contiguous spread from nasal or paranasal cavities to adjacent structures like orbit, optic chiasma, meninges, and pituitary gland. The second is vasculitis affecting both intracerebral or spinal cord vessels, and the least common mechanism is the remote intracerebral granulomatous lesion [7]. The relevance of accelerated atherosclerosis can be seen in the review of case reports given below, depicting patients of different demographics presenting with ischemic stroke and later developing other features of WG (Table 1).

**Table 1:** A review of previous case reports published on autoimmune-associated vasculitis presenting as ischemic stroke NIHSS: National Institutes of Health Stroke Scale; MRI: magnetic resonance imaging; ANCA: anti-neutrophil cytoplasmic antibody; PR3: proteinase 3; ESR: erythrocyte sedimentation rate; CRP: C-reactive protein; ANA: anti-neutrophil antibody; IV: intravenous; TPA: tissue-plasminogen activator

Ref. number	Age and sex	Clinical features	NIHSS score	MRI findings	Serology	Renal and/or pulmonary involvement at the time of presentation	Treatment	Outcome
[8]	41/M	Weakness and numbness of right hand and foot, right facial paresis, nausea, vomiting, dysarthria	3	Acute ischemic infarct in the right caudal lateral medulla of the brainstem	Increased C-ANCA/anti-PR3, and positive rheumatoid factor (RF). Elevated ESR, CRP, and platelets; ANA and P-ANCA were negative	Diffuse, necrotizing crescentic glomerulonephritis of pauci-immune type	Methylprednisolone, cyclophosphamide, hemodialysis, aspirin	Two weeks later, no focal neurological deficits and remained in remission
[9]	48/M	Right hemiparesis, diffuse arthralgias (especially left ankle)	-	Ischemic stroke in the deep left Sylvian fissure	Elevated C-ANCA/anti-PR3, elevated CRP	No pulmonary or renal involvement on initial presentation	Monthly cyclophosphamide bolus, corticosteroids	After one month, the development of vascular purpura, renal damage, purulent rhinitis, and oral ulcers
[10]	58/M	Hemiparesis, dysarthria, facial droop, fever, myalgias, dark urine, urticarial rash over the trunk and extremities	4	Left internal capsule infarct and multiple punctuate infarcts bilaterally	Elevated C-ANCA, ESR, CRP, and negative P-ANCA	Renal failure, no pulmonary involvement	Corticosteroid, rituximab	Developed multiple intracranial hemorrhages after given IV TPA, marked improvement after three months
[11]	54/F	Left-arm weakness with flu-like symptoms worsening to weakness in the entire left side of the body with inattention	2	Small infarct in the posterior limb of the internal capsule with hemorrhagic transformation and intraventricular extension	Positive anti-PR3	None involved	IV cyclophosphamide and prednisolone	The patient made considerable progress
[12]	42/M	Right sensorimotor hemisyndrome accompanied by motor aphasia	-	Hemorrhagic stroke in the left basal ganglia, without underlying ischemia	Elevated C-ANCA/anti-PR3	None involved		Six months later the patient developed acute respiratory distress, oral ulcers, epistaxis, and arthralgias
[13]	52/F	Dizziness, ataxia, left ophthalmoplegia, right facial paresis, left hemi-sensory loss	-	Ischemic stroke in the right medulla oblongata, with multiple infarcts in white matter	Elevated C-ANCA/anti-PR3, ESR, CRP, and thrombocytosis	None on initial presentation	-	One month later, fatigue, weight loss, acute kidney failure

CNS vasculitis is a diagnostic challenge as conventional imaging is not suitable for small vessel vasculitis, and taking a biopsy is often not possible [14]. Digital subtraction angiography is one of the gold standard investigations for vasculitis. In the case of large-vessel involvement of the brain, angiography shows alteration consistent with vasculitis in nearly 90% of the affected patients [15]. Nevertheless, in AAV-associated CNS disease, the diameter of small vessels (50-300 micrometers) is below the threshold for routine angiography [5].

CNS vasculitis is managed with high-dose corticosteroids. Cyclophosphamide in combination with steroids has proven beneficial in pachymeningitis and orbital granuloma only [16]. Sometimes, plasmapheresis is added to prevent relapses in maintenance therapy [5]. A decrease in C-ANCA is a valid marker for monitoring the response of the treatment. Early treatment directly correlates with a more significant reduction in neurological complications and prevents long-term sequelae [5].

## Conclusions

Small vessel vasculitis can present with ischemic stroke with hemorrhagic transformation in the early stages, and its etiology can often be missed if no other finding of AAV is present. Young patients with stroke have a better chance of recovery than the older population. However, the immense psycho-social, economic, and functional burden on the patients and their family members are evident for years after an episode. The situation becomes even grimmer if they are the sole providers and general income earners of the family, which significantly impedes their quality of life. Hence, identifying the risk factors of the said pathology is of paramount importance for preventing such debilitating complications.
